# The Suicide Rate in the Elderly Population of Iran between 2008 and 2014

**DOI:** 10.34172/jrhs.2020.06

**Published:** 2020-02-24

**Authors:** Dinaz Razai, Mohammad Reza Ghadirzadeh, Seyed Amirhosein Mahdavi, Jalil Hasani, Seyed Saeed Hashemi Nazari

**Affiliations:** ^1^School of Public Health and Safety, Shahid Beheshti University of Medical Sciences, Tehran, Iran; ^2^Legal Medicine Research Centre, Legal Medicine Organization of Iran, Tehran, Iran; ^3^Department of Public Health, Torbat Jam Faculty of Medical Sciences, Torbat Jam, Iran; ^4^Safety Promotion and Injury Prevention Research Center, Department of Epidemiology, School of Public Health and Safety, Shahid Beheshti University of Medical Sciences, Tehran, Iran

**Keywords:** Suicide, Elderly, Iran

## Abstract

**Background:** We aimed to investigate the suicide rate led to death in the elderly population of Iran between 2008 and 2014.

**Study design:** A cross-sectional study.

**Methods:** The present study was conducted on all suicide-related deaths in elderly people (≥65 yr) during the years 2008 to 2014 reported to the Iranian Legal Medicine Organization. For data collection, legal medicine standard form was used and the cases were classified by age, gender, suicide way and time (year). The incidence of death from suicide was calculated by age and sex. Statistical soft-ware stata12 was used to analyze data. The significance level has been considered to be 0.05.

**Results:** Overall, 1,601 suicide-related deaths were investigated throughout the country. The mean age was 70.36 ± 0.17 years. The incidence trend (per 100,000 people) of the elderly suicides in Iran indicates that successful suicides have been on the rise, rising from 3.7 in 2008 to 4.37 per 100,000 people in 2014.

**Conclusions:** It is necessary to identify and treat suicidal important predisposing factors of suicide such as psycho-social illnesses including depression and also implement prevention programs and policies for this fast-rising population age-group.

## Introduction

Suicide is one of the major causes of abnormal death in the world, and it is the fifth leading cause of death in 65 years old and more. Suicide is responsible for 1.4% of deaths in all over the world^1,2^. According to the World Health Organization (WHO) reports, there were an estimated 793000 suicide deaths worldwide in 2016. This indicates an annual global age-standardized suicide rate of 10.5 per 100000 population ^3^. The incidence rate was more in low and middle income countries than in high income countries ^4^. Suicide does not just occur in high-income countries, but is a global phenomenon in all regions of the world. In fact, over 79% of global suicides occurred in low- and middle-income countries in 2016 ^5^. Approximately 60% of the suicides occur in Asian countries. It seems that suicide has come from Western Europe to Eastern Europe, and now Asia has become as the heart of the problem^1,6,7^.

Despite the low overall incidence in some countries or the low incidence in the youth and adolescents group, the incidence of suicide in the people at the age of 50 years in some countries is considered to be a major health problem. A multi-centered study (in 13 European countries) conducted by the World Health Organization and Europe on suicidal behavior showed that the average suicide rate among the elderly over 65 was 29.3 per 100,000 and suicide attempt rate was 61.4 per 100,000. Also, in addition to the high suicide attempt rate in this age group, the proportion of suicide attempt to successful suicide rate in this group was very close and almost two to one^8,9^. Although the suicide-related deaths in some countries have been declining, there has been a growing trend in the elderly ^9^.

According to a WHO report in 2012, in Iran the rate of suicide is 5.3 per 100,000 people. The total number of cases was 4068, of which 66% occurred in men. The highest incidence of suicide in both sexes was in the age group of 70 years and over (16.1 per 100,000) and then in the age group of 15-29 years old (7.8 per 100,000) and the lowest incidence were reported in the age group of 5-14 years old (0.7 per 100,000) and then, in the age group of 50-69 years old (5 per 100,000) ^1^.

Some of the most important risk factors for suicidal deaths in the elderly are mental disorders, especially depression, physical illnesses, longevity, having suicidal attempts, and lack of social support as well as personality traits ^10^. In high-income countries, more than 90% of suicides have some degree of mental disorders, and in Asian countries such as China and India, about 60% of suiciders have suffered from psychiatric disorders ^1^. The most commonly ways of suicide include poisoning, hanging and suicide with weapons. Of course, the choice of suicide is different base on demographic groups^1,11,12^. Studies have shown that factors such as age, gender, and education level are important factors in choosing the suicide way in Iran^1,13^.

Studies on suicide in the elderly population have been widely reported in industrialized and developed countries, but little attention is paid to this in less developed and non-industrialized countries. As a result, there is a huge gap in our knowledge about risk factors that are associated with this phenomenon. In Iran, there is a lack of studies on suicide in the elderly. Indeed, a significant shift in attention to suicide in the elderly have not been seen despite the general growth of university research on suicide in Iran ^14-17^. Therefore, this study was designed to investigate successful suicide rate in the subgroup of elderly in the whole country from 2009 to 2015 that with the help of this data it is possible to provide valuable information for the need assessment, design and revision of the country's development plans, and indicators.

## Methods

The present study was a cross-sectional, descriptive-analytic study. This research includes all cases of suicide that resulted in death in the elderly, which was reported to the legal medicine organization in all provinces from 2008 to 2014. For data collection, legal medicine standard form was used and the cases were classified by age, gender, suicide way and time (year).

According to the Iranian's law, every suspicious death should be investigated by legal medicine organization and certificate of these deaths must be issued just by this organization. So it is expected that almost all suicides occurred in the country, be referred and registered. Hence the suicidal database of this organization is the most complete source for suicide mortality data. In this study, International Classification of Disease, 10th edition (ICD-10) codes X60-84 is assigned for classification of deaths due to suicide as the external cause of death. Since the outcome was count, the Poisson model was used to analyze the data. Primary outcome is count of suicide or suicide number and all of cases are fatal suicides.

This study was registered at Legal Medicine Research Center in Iran and was approved by the Ethics Committee of the Legal Medicine Organization (IR.LMO.REC.1398.018).

The data obtained from the legal medicine organization were carefully considered for the duplication and accuracy of the registration of variables related to this study. In order to calculate the incidence of suicide by age and sex in the years 2008 to 2014, the number of deaths of the elderly people in each age and sex group were divided by the population of each age and sex group, which were obtained from the Center of Statistics of Iran, and eventually the incidences per one hundred thousand people were reported. Age group was divided into three groups: the young-elderly aged 60 to 74 years old, the middle-elderly aged 75-84 and old-elderly aged 85 years and more^18,19^. Statistical soft-ware stata12 was used in this study to analyze the data. To determine the relationship between suicide and gender, age (age groups), Poisson regression was used. To evaluate changes in incidence rate trend, the Cochran-Armitage linear trend test was used. In this study, the significance level was considered to be less than 0.05.

## Results

Overall, 1,601 cases of suicide-related deaths were investigated throughout Iran. The mean age was 70.36 ±0.17 years. Of all cases, 76% were male (1217 cases) and 24% were female (384 cases). 50.5% of suicidal cases were in the age group of 60-74 yr, and the lowest suicide percentage (9.9%) occurred in the age group above 85 years (Table 1). The suicide rate in men was more than three times that of women, which this ratio was statistically significant (Rate Ratio: 3.57, CI: 2.72, 4.67). Most suicides in men were by hanging and then poisoning, and in women, the majority of suicides were done using hanging and self-immolation way. A statistically significant difference was observed between suicide methods in men and women (*P* <0.001) (Table 2).

**Table 1:** Epidemiologic characteristics of elderly successful suicides reported from Legal Medicine Organization of the country in the years 2008 to 2014

**Year** **No. of Suicides** **Variables**	**2008**	**2009**	**2010**	**2011**	**2012**	**2013**	**2014**	**Total**
	**202**	**196**	**209**	**194**	**236**	**264**	**300**	**1601**
	**n (%)**	**n (%)**	**n (%)**	**n (%)**	**n (%)**	**n (%)**	**n (%)**	**n (%)**
Age (yr)								
60-74	139 (68.80)	70 (71.93)	69 (73.68)	47 (64.94)	62 (63.98)	63 (62.87)	63 (74.00)	513 (50.50)
75-84	52 (25.74)	49 (25)	44 (21.05)	56 (28.86)	69 (29.23)	72 (27.27)	60 (20.00)	402 (39.60)
≤85	11 (5.44)	6 (3.06)	11 (5.26)	12 (6.18)	16 (6.77)	26 (9.84)	18 (6.00)	100 (9.90)
Sex								
Male	148 (73.26)	147 (75.00)	162 (77.51)	152 (78.35)	180 (76.27)	196 (74.24)	232 (77.33)	1217 (76.00)
Female	54 (26.73)	49 (25.00)	47 (22.48)	42 (21.64)	56 (23.72)	68 (25.75)	68 (22.66)	384 (24.00)
Suicide method								
Hanging	111 (54.95)	97 (49.48)	121 (57.89)	111 (57.21)	116 (49.15)	113 (42.8)	155 (51.66)	824 (51.50)
Self-immolation	41 (20.29)	31 (15.81)	32 (15.31)	15 (7.73)	27 (11.44)	40 (15.15)	28 (9.33)	214 (13.40)
Poisoning	30 (14.85)	36 (18.36)	33 (15.78)	41 (21.13)	54 (22.88)	62 (23.48)	52 (17.33)	308 (19.20)
Another	17 (8.41)	29 (14.79)	20 (9.56)	24 (12.37)	39 (16.52)	48 (18.18)	65 (21.66)	242 (15.10)
Unknown	3 (1.48)	3 (1.53)	3 (1.43)	3 (1.54)	0 (0.00)	1 (0.37)	0 (0.00)	13 (0.08)

**Table 2:** Determining the relationship between gender and age with suicide methods

**Suicide**	**Incident Rate Ratio (95% CI)**	***P*** **value**
Sex		
Male	3.57 (2.72, 4.67)	0.001
Female	1.00	
Age group		
60-74	1.05 (0.65, 1.70)	0.830
75-84	0.89 (0.52, 1.51)	0.670
≥85	1.00	

**Figure 1:**
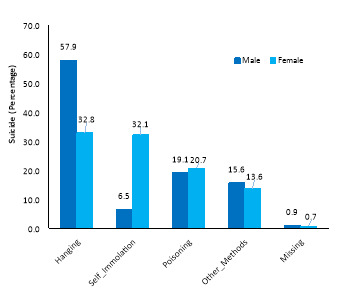
Relative distribution of suicide-related deaths in the Iranian elderly population between 2008 and 2014 by of suicidal method and gender

Figure 1 shows the relative distribution of each suicide method by sex. Most suicides have occurred in men and women due to hanging.

Cochran-Armitage linear trend test showed that the trend of changes in incidence rate in Iran as well as in subgroups of men and age group of 64-75 yr was significant during the 7 yr of study (*P* <0.05) (Table 3). To determine the relationship between suicide and gender and (age groups) Poisson regression was used. In this analysis, the age variable was divided into three subgroups of 60-74, 84-75 and ≥85. The results of Poisson's regression analysis on data in 2014 showed that the relationship between sex variable and suicidal death in the elderly was statistically significant (*P* <0.001). But there was no significant relationship between age and suicide (*P* >0.05). The results of Poisson's regression analysis showed that the rate of suicide in men is approximately twice that of women. IRR = 1.93 (95% CI: 1.33, 2.79).

**Table 3:** The investigate the trend changes in the incidence rate with Cochran-Armitage linear trend test by sex and age group

**Variables**	**2008**	**2009**	**2010**	**2011**	**2012**	**2013**	**2014**	**Chi** ^2^ **for trend**	***P*** **value**
Sex									
Male	5.32	5.15	5.53	5.03	5.74	6.03	6.87	8.95	0.003
Female	2.00	1.73	1.58	1.34	1.72	2.02	1.95	0.38	0.530
Total	3.70	3.46	3.56	3.15	3.70	3.99	4.37	7.36	0.007
Age group (yr)									
60-74	3.49	3.45	3.67	2.91	3.34	3.51	4.48	4.05	0.044
75-84	4.12	3.69	3.16	3.80	4.65	4.81	3.98	1.45	0.230
≥85	4.42	2.14	3.50	3.35	4.29	6.67	4.42	3.07	0.080
Total	3.70	3.46	3.56	3.15	3.70	3.99	4.37	7.36	0.007

## Discussion

The present study evaluates the rate of successful suicide in the elderly population (over 60 years old) in Iran during the years 2008 to 2014. The trend of successful suicide was decreasing between 2008 and 2011, but from 2011 to 2014 has been increasing with a smooth slope. In sum, the rate of successful suicide has increased slightly from the beginning to the end of the study period (2008 to 2014). The incidence rate in women over the course of seven years has been roughly constant, but the suicide rate in men has risen sharply since 2011 with a steep slope. In the study of Hajabi and colleagues during the years 2009 to 2012 in the country, suicide attempt rate in people over 65 years old has grown around 12% ^20^. The findings of this study were in line with the study of Lee et al in South Korea. The incidence of suicide in South Korea has risen from 10.7 per 100,000 people in 1993 to 21.9 per 100,000 people in year 2016. By 2011, the suicide rate has been increasing, but after that it has been decreasing till 2016 ^21^. Contrary to the results of the present study, the incidence of suicide in the elderly over the age of 60 years in China has declined from 1987 to 2014 ^22^. Significantly, the increasing incidence of suicide in the elderly can be attributed to the aging of the communities and the growing population of this age group. In China, the elderly population is 8.9 percent of the country's population, but 38.2 percent of all deaths attributed to suicide belong to this age group ^23^. Other reasons include increasing the prevalence of physical and mental illnesses, fundamental changes in the community and the family, as well as changing the cultural foundations and the weakening of the family foundation, as well as changing the community and family customs in the country over the last years.

In this study, men have attempted suicide more than three times than women. According to the World Health Organization, in 2016, the death rate for suicide in men has been about twice that of women ^24^. In the Zhong study, the male to female sex ratio was 1.4. In other words, suicide in older men in China has been reported 40 percent higher than in women in the period 2013-2014 ^23^. In the study of Pinto et al., Who had evaluated the suicide rate in the elderly in Brazil from 1980 to 2009, the sex ratio was 4.1 times in 1980 and 5.3 times in 2009 ^25^. In the study of Sharafkhani et al., the ratio of the chance of suicide led to death in men was 3 times higher than that of women ^24^. In most studies, suicide attempts have been more prevalent in women and successful suicides were more prevalent in men. One of the main reasons for the big difference of suicide rate in men compared to women is the use of harsher methods such as the use of hanging method and firearms that are fatal and the likelihood of survival is low. In contrast, women tend to use poisoning with drugs and chemicals that are less considered to be fatal, and they are likely to survive ^11^. The use of aggressive and violent methods of suicide increases the risk of successful suicide 12 times ^24^. Other reasons for more suicide among men include drug addiction and subsequent mental-neurological diseases in men ^26^. According to research results, the likelihood of successful suicide in people with a history of mental-neurological diseases is 5 times higher than those without a history of psychiatric disorders ^24^.

Other findings in this study show that the highest suicide rate in the elderly is between the ages of 60 and 74 and the lowest suicide rate is in the age group of 85 years and more. Consequently, in the current study, with increasing age, suicide has been decreasing. The findings of this study contradicted the results of Zhong et al. ^23^ In that study, the death rate had a rising trend and had reached a peak in the 85-year-old group. In most parts of the world, the risk of death from suicide has also increased with the increase in age. In the study of Lee et al., both in the early years of the study (1993) and the ending year of the study (2016), the rate of suicide has increased as the age has increased. Suicide rate in the age group of 70 years or more had also an increasing trend in contrast to the age group of 10 to 19 years ^21^. According to reviewed research results, in most parts of the world, the highest proportion of suicide has occurred in the elderly over the age of 70 ^21^. In Sang Oh et al. study on suicidal methods, it was found that in the people over 65 years of age, high-lethal methods with low survival rates were used compared to young people ^27^. Another point is that suicide has been reported less frequently in elderly than teenagers and young people, but successful suicide is much higher in elderly than in youth ^21^ Significantly, the increase in suicide rate in the elderly can be attributed to disability, constraint of movement and sedentary life, poor self-care and high prevalence of chronic physical and psychological disorders in this group. From the other reasons of increasing suicide in the elderly one can point to factors such as loneliness duo to (spouse death or divorce), unemployment, and low educational level. In a study by Shao Y and colleagues in Shanghai, China, successful suicidal people with a history of mental illness were more likely to be over the age of 65 years and individuals with successful suicide that had no history of mental illness were more likely to be in the age group of less than 65 ^28^. In the present study, the suicide rate in the age group of 60-74 years old has increase during the study period but in other elderly age groups have been almost constant.

In the present study, hanging methods have been reported in both women and men as the most common method of suicide. The findings of this study were consistent with the results of Hajebi study and the hanging method in suicide cases in Iran during the years 2009 to 2013 was the most common method of suicide ^20^. In other studies in Iran, hanging is the deadliest method of suicide ^20^. In the study of Oh et al., the use of hanging method, drowning and firearms method, which are considered to be high traumatic methods, included only 16% of the all the methods which were used to attempt to suicide, but 78% of suicide deaths were done by these methods ^27^. Due to the physical condition and mental conditions of the elderly, as well as the simplicity and availability and high fatality of hanging method, it is a highly chosen option for suicide in the elderly. On the other hand, this method is easily identifiable and reported. In America, because of access to firearms, they are the most common methods of suicide ^27^. However, unknown causes may also play an important role in choosing suicide method ^29^.

The most important strengths of this study are the following: (a) Accessing to the country data without using any sampling. (b) Using the most complete, accurate and reliable data on successful suicides in the country (according to the law, all suspected deaths must be confirmed by Legal Medicine Organization, as a result, the most comprehensive source of data is expected to be used in this research). (c) Precise trimming of the data in terms of the studied variables, separating suicide data from other causes of death and eliminating duplicates of data.

As with other similar studies, the following limitations can be noted in the present study: (a) Lack of access to data of suicide attempts throughout the country and to compare its epidemiological characteristics with successful suicides. (b) Probable low reporting (due to social stigma and legal issues) in suicide data. (c) Lack of access to data on other factors affecting the suicidal phenomenon, such as: mild and severe mental disorders, social indicators (employment status, drug addiction, substance abuse and alcohol consumption), and socioeconomic indicators and other factors affecting suicide in the whole country.

## Conclusion

The suicide rate in the elderly not only has not been decreased in the past years but while maintaining the trend of the past few years; bit it has also grown a little. On the other hand, due to increased life expectancy, the proportion of elderly people will increase significantly over the coming decades. Therefore, it is necessary to identify and treat suicidal important predisposing factors of suicide such as psycho-social illnesses including depression and also implement prevention programs and policies for this fast-rising population age-group. One of the medical services of the medical universities in the provision of health services to the elderly, and on the other hand, it is also a psychologist in every health center. Therefore, it is possible to apply the existing potential for preventing suicide in the elderly. Attention to socio-economic issues of the elderly is also a major factor in tackling the increasing growth in suicide in the elderly, which should be considered by policy-makers in the community.

## Acknowledgements

We would like to appreciate all the staffs of Iran’s LMO and Legal Medicine Research Center and also all those researchers who helped us conduct this study.

## Conflict of interest

The authors declare that there is no conflict of interests.

## Funding

This study is financially supported by the Shahid Beheshti University of Medical Sciences and also Legal Medicine Organization of Iran.

## Highlights

- The incidence trend of the elderly suicides in Iran rising from 3.7 in 2008 to 4.37 per 100,000 people in 2014.
- The suicide rate in men was more than three times that of women.
- Cochran-Armitage linear trend test showed that the trend of changes in incidence rate in Iran as well as in subgroups of men and age group of 64-75 yr was significant during the 7 years of study.
